# Emotional Word Processing in Patients With Juvenile Myoclonic Epilepsy

**DOI:** 10.3389/fneur.2022.875950

**Published:** 2022-06-01

**Authors:** Lucas Johannes Rainer, Martin Kronbichler, Giorgi Kuchukhidze, Eugen Trinka, Patrick Benjamin Langthaler, Lisa Kronbichler, Sarah Said-Yuerekli, Margarita Kirschner, Georg Zimmermann, Julia Höfler, Elisabeth Schmid, Mario Braun

**Affiliations:** ^1^Department of Neurology, Christian-Doppler Medical Centre, Paracelsus Medical University, Centre for Cognitive Neuroscience Salzburg, Member of the European Reference Network, Epicare, Salzburg, Austria; ^2^Neuroscience Institute, Christian-Doppler Medical Centre, Paracelsus Medical University, Centre for Cognitive Neuroscience, Salzburg, Austria; ^3^Department of Psychiatry, Psychotherapy & Psychosomatics, Christian-Doppler Medical Centre, Paracelsus Medical University, Salzburg, Austria; ^4^Department of Psychology, Naturwissenschaftliche Fakultaet, Centre for Cognitive Neuroscience, Paris-Lodron University, Salzburg, Austria; ^5^Department of Public Health, Health Services Research and Health Technology Assessment, UMIT–University for Health Sciences, Medical Informatics and Technology, Hall in Tirol, Austria; ^6^Karl-Landsteiner Institute for Neurorehabilitation and Space Neurology, Salzburg, Austria; ^7^Department of Mathematics, Paris-Lodron University, Naturwissenschaftliche Fakultaet, Salzburg, Austria; ^8^Team Biostatistics and Big Medical Data, IDA Lab Salzburg, Paracelsus Medical University, Salzburg, Austria; ^9^Research and Innovation Management, Paracelsus Medical University, Salzburg, Austria

**Keywords:** discrete emotion, dimensional emotion, neuropsychology, juvenile myoclonic epilepsy (JME), implicit emotion processing

## Abstract

**Objective:**

According to Panksepp's hierarchical emotion model, emotion processing relies on three functionally and neuroanatomically distinct levels. These levels comprise subcortical networks (primary level), the limbic system (secondary level), and the neocortex (tertiary level) and are suggested to serve differential emotional processing. We aimed to validate and extend previous evidence of discrete and dimensional emotion processing in patient with juvenile myoclonic epilepsy (JME).

**Methods:**

We recorded brain activity of patients with JME and healthy controls in response to lexical decisions to words reflecting the discrete emotion fear and the affective dimension negativity previously suggested to rely on different brain regions and to reflect different levels of processing. In all study participants, we tested verbal cognitive functions, as well as the relationship of psychiatric conditions, seizure types and duration of epilepsy and emotional word processing.

**Results:**

In support of the hierarchical emotion model, we found an interaction of discrete emotion and affective dimensional processing in the right amygdala likely to reflect secondary level processing. Brain activity related to affective dimensional processing was found in the right inferior frontal gyrus and is suggested to reflect tertiary level processing. Psychiatric conditions, type of seizure nor mono- vs. polytherapy and duration of epilepsy within patients did not have any effect on the processing of emotional words. In addition, no differences in brain activity or response times between patients and controls were observed, despite neuropsychological testing revealed slightly decreased verbal intelligence, verbal fluency and reading speed in patients with JME.

**Significance:**

These results were interpreted to be in line with the hierarchical emotion model and to highlight the amygdala's role in processing biologically relevant stimuli, as well as to suggest a semantic foundation of affective dimensional processing in prefrontal cortex. A lack of differences in brain activity of patients with JME and healthy controls in response to the emotional content of words could point to unaffected implicit emotion processing in patients with JME.

## Introduction

A multitude of neuroimaging and electrophysiological evidence has been collected in recent years on emotional processing in the human brain (1). However, it is still not entirely clear which brain regions reflect emotional processing and what is the time course of such processing (2). Two main views provide evidence on emotional processing in humans. Discrete emotion theories (3–5) suggest that a limited number of discrete emotions (e.g., anger, fear, sadness) are evolutionary ingrained (3) and culturally universal (6). In contrast, dimensional emotion theories (7, 8) propose that a limited number of affective dimensions (e.g., valence and arousal) constitute the basis of emotional processing (9). Initially seen as opposing views, recent evidence is in support of the idea that discrete emotions and affective dimensions could both reflect emotional processing in humans (10, 11). A framework which is in support of this way of processing is provided by the hierarchical emotion model of Panksepp (5, 12, 13). According to this model, discrete emotions and affective dimensions are not seen as opposing views, but rather as describing different processes operating on different, neuroanatomically distinguishable levels (14). At a primary process level, discrete emotions are thought to originate in subcortical circuits, such as the periaqueductal gray (PAG). At a secondary process level, primary process level emotions are transformed into conditioned responses by classical and instrumental conditioning, which is served by limbic structures including the amygdala (15, 16). At the tertiary process level, neocortical structures such as the prefrontal cortex are thought to interact with the lower process levels by cognitive processes and to be shaped by socio-cultural demands clustering primary emotion information further into constellations of positive and negative affect (17, 18). Reading single words with emotional content has been reported to activate brain areas such as the hippocampus, parahippocampal gyrus, amygdala, anterior and posterior cingulate cortex, and orbitofrontal cortex (19–23). Briesemeister et al. (10, 11) reported electrophysiological and neuroimaging evidence for the processing of discrete emotion words reflecting high and low happiness but also for the processing of words reflecting the affective dimension of positivity in line with Panksepps hierarchical emotion model. The electrophysiological results indicated sequential processing of emotion information, with the discrete emotion happiness, affecting the early visual N1 component, and the affective dimension positivity, reflected in an N400-like component and the late positive complex. (10). Neuroimaging revealed limbic activity in the right amygdala for words reflecting the discrete emotion happiness, and activity in the prefrontal cortex such as the left and right inferior frontal gyri and left medial frontal gyrus for the affective dimension of positivity. These and other results strongly suggest that emotion processing relies on extended networks and might be altered if structures like the limbic system or the prefrontal cortex are impaired. This could be observed in certain neurological conditions, such as epilepsy in which patients have impaired emotion recognition (24–30). Juvenile myoclonic epilepsy (JME) comprises 5–10% of all epilepsies (31) and is one of the most common age-related idiopathic generalized epilepsies with a high reported genetic pre-disposition (32). JME-onset peaks between 14 and 16 years, usually presents massive myoclonic, generalized tonic-clonic and absence seizures (33–36). Executive functions are reported to be impaired in JME, comprising mental flexibility, inhibition of automated reactions, abstraction and categorization, planning and verbal fluency (37–40). Behavioral problems in patients with JME, such as poor social adjustment and impulsive personality traits, resembling patients with frontal lobe damage, are often observed (41, 42). The prevalence of psychiatric disorders of patients with JME varies between 35 and 49% and studies demonstrated increased mood, anxiety, and cluster B personality disorders (41, 43). Neuroimaging revealed subtle structural and functional alterations in thalamus and frontal cortex associated with cognitive, behavioral and emotional disturbances (44–47). Furthermore, recent studies reported morphological and functional alteration involving thalamo-cortical circuits, cerebellum, bilateral hippocampi, and cingulate, insular and occipital cortices (48–50). Thus, the clinical picture of patients with JME shows cognitive as well as emotional impairments.

In this prospective fMRI study on patients with JME and healthy controls we aimed to extend and to validate the previous reported dissociation of brain regions involved in discrete and dimensional emotion processing (11), by presenting words reflecting the negative emotion fear and the affective dimension of negativity. In line with the results of Briesemeister et al. (47) we hypothesized that the processing of words with high fear values differs from those with low fear values in the right amygdala and that the inferior frontal cortex shows increasing activity with increasing negativity of words. Furthermore, we expected general faster response times for words with high fear values compared to words with low fear values. In addition, we expected patients with JME and healthy controls to differ in the processing of words reflecting the affective dimension of negativity and that this differential processing is revealed by differences in brain activity in the inferior frontal cortex, since neuropsychological and neuroimaging evidence showed altered executive functioning in patients with JME. Concerning the neuropsychological testing, we expected patients with JME to show deficits in several verbal and cognitive measures related to executive functions and to be at higher risk of experiencing psychiatric disorders.

## Materials and Methods

### Participants

A total of 47 patients with JME were recruited in the Epilepsy Outpatient Clinic, Clinical Department of Neurology, Paracelsus Medical University, Salzburg, and were compared to 62 gender-, education- and age-matched healthy controls. Inclusion criteria for patients comprised the diagnosis of JME based on criteria of the International League against Epilepsy (51), above 14 years of age with willingness to participate in the project as well as informed consent obtained from patients or parents. Exclusion criteria for patients comprised the occurrence of any epileptic seizure <72 h prior to fMRI, neurological illnesses other than JME, structural lesions (if known from previous preliminary examinations), incompatibility with MRI investigation (e.g., metal implants, claustrophobia), pregnancy, and acute intake of Benzodiazepines. Exclusion criteria for healthy controls comprised individuals <18 years of age, previously known psychiatric and neurological illnesses or known structural lesions of the brain, incompatibility with MRI investigation, and pregnancy. Mean onset of epilepsy was at 14.23 years of age, mean epilepsy duration was 12.85 years. Three patients (6.2%) did not receive any antiseizure medication, 28 patients (58.3%) received monotherapy and 17 patients (35.4%) polytherapy. For a detailed sample description see **Table 2**. All participants gave written informed consent in accordance with the guidelines set by the local Ethics Committee.

### Methods

#### Procedure

Psychiatric and neuropsychological assessments and functional magnet-resonance-imaging (fMRI) were carried out after all participants gave written informed consent. The test battery comprised a structured clinical interview on psychiatric and personality disorders, verbal intelligence, verbal fluency, verbal memory and reading speed. As the entire battery took about 2 h to complete, participants who were not able to complete it at once and were allowed to take part in two sessions. Additionally, participants completed structural and functional MRI. fMRI lasted about 1 h. fMRI was performed either before or after neuropsychological evaluation, or if participants wished to do so, on one of the following days. Healthy controls were compensated €100 for their participation, patients with JME did not receive financial compensation.

#### Neuropsychological and Clinical Tests

##### SCID I + II

The Structured Clinical Interview for Axis I disorders (SCID I) (52) and for Axis II disorders (SCID II) (53), based on the Diagnostic and Statistical Manual of Mental Disorders-IV, are considered to be the gold standard for a semi-structured assessment of clinical disorders and personality disorders, respectively. German versions (54) were used to assess DSM-IV Axis I and Axis II pathology.

##### Multiple-Choice Vocabulary Test

The Mehrfach-Wortschatz-Intelligenzttest (MWT-B) is a multiple-choice vocabulary test which serves as an estimate of crystalline verbal intelligence (55, 56). In this test the participants are asked to identify a known colloquial or scientific word in-between four non-words. The 37 items are arranged by increasing difficulty.

##### Verbal Fluency Test

The Regensburger Wortflüssigkeitstest (RWT) (57) assesses verbal fluency. Specifically, we measured semantic fluency by counting the number of animals and the number of - alternating – fruits and sports, as well as lexical fluency of words starting with the letter “S” and, again alternating, words starting with “G” and “R.” For each condition, the participant was given 2 min time.

##### Auditory Verbal Learning Test

The Verbaler Lern- und Merkfähigkeitstest (VLMT) (58, 59) is a German version of Rey's (60) Auditory Verbal Learning Test (61). The VLMT consists of fifteen words, which are spoken by the examiner on five successive trials. Following each trial, the individual recalls all memorized words. After the five trials, a second group of words (distraction) is read aloud and recalled by the individual. After the distraction list, the individual recalls the words from list one. Following approximately 20 to 30 min, the individual again recalls all words from list one. Finally, the individual is instructed to recognize words from list one from a list of forty-five words. The test measures verbal learning ability, verbal memory consolidation and verbal recognition.

##### Sentence Reading Test

The sentence reading test, developed by Bergmann and Wimmer (62) requests participants to read short sentences and judge their semantic content. The sentences are of simple content so that erroneous markings are infrequent and the number of sentences marked within 3 min largely reflects reading speed and semantic comprehension.

#### fMRI Paradigm

##### Lexical Decision Task (LDT)

When reading single words in the fMRI-scanner, participants completed a lexical decision task (LDT) (63) in which subjects were shown German nouns (targets; e.g., Henker [hangman]) or German non-words (non-targets; e.g. Luicke). Participants had to decide whether the presented stimulus was a correctly spelled German word or not. Non-words were created changing two letters of an existing German noun. The LDT is the most used task to measure lexical access to the visual word form (63–66).

##### Stimuli

Following the design of Briesemeister et al. (10, 11), we used 120 German four- to eight-letter nouns and 60 non-words in a 2 (discrete emotion) x 2 (affective dimension) within subjects design with 30 items per cell. Contrary to Briesemeister et al. who used happiness and positivity, we tried to extend previous results by using words reflecting the emotion fear and words reflecting the affective dimension of negativity. Stimuli were chosen from the Berlin Affective Word List-Reloaded (67) (BAWL-R) and its discrete-emotion extension, the Discrete Emotion Norms for Nouns BAWL (68) (DENN-BAWL). The BAWL-R is a rating-based word list with norms for affective valence (7-point Likert scale, ranging from negative [-3] to positive [3]) and arousal (5-point Likert scale, ranging from low [1] to high arousing [5]). Given that negativity judgments and BAWL-R's valence ratings are highly correlated (69), words with BAWL-R scores between −0.7 and 0.7 were defined as being of neutral valence (NEU), and words with valence scores below 1 were defined as being of negative valence (NEG). The DENN-BAWL norms were used to classify words as being either strongly or not strongly related to fear, with low-fear words (lowFEAR) having DENN-BAWL scores below 2.0 and high-FEAR words (highFEAR) having scores above 2.0. DENN-BAWL norms indicate the extent to which a single word is related to one of five discrete-emotion categories, with high scores indicating a strong relation. The resulting four orthogonal conditions (lowFEAR + NEU: e.g., “AGENT,” Engl. “AGENT”; lowFEAR + NEG: e.g., “MIETE,” ENGL “RENT”; highFEAR + NEU: e.g., “MESSER,” ENGL. “KNIFE”; highFEAR + NEG “TEUFEL,” ENGL. “DEVIL”) were uncorrelated for fear and valence scores, indicating that lowFEAR + NEG words are perceived as being negative but not relate to the discrete emotion fear, and vice versa. For statistical details about the stimulus set, see Table 1. Mean levels of arousal, imageability, orthographic neighborhood size, frequency of orthographic neighbors, frequency of higher-frequent orthographic neighbors, and the mean number of letters, syllables, bigram frequency, and higher-frequency orthographic neighbors were controlled using analyses of variance (ANOVAs, all *F*s >1; see Table 1). The psycholinguistic variables were matched for the highFEAR-versus-lowFEAR and NEU-versus-NEG contrasts and tested with pairwise *t*-tests (all *t*s <1). A list containing all 120 words and 60 non-words is provided in the Supplementary Materials. In addition, 30 null-events, serving as baseline, in the form of a fixation cross (“+”) were included, which was meant to increase the signal-to-noise ratio of the fMRI paradigm.

**Table 1:** Descriptive statistics of the stimulus set, along with the behavioral responses.

	**lowFEAR + NEU**	**lowFEAR + NEG**	**highFEAR + NEU**	**highFEAR + NEG**	***F-v*alue**	***p-v*alue**
*Fear*	1.5 (0.26)	1.5 (0.24)	2.2 (0.29)	2.4 (0.34)	84.232	<0.001
*Negativity*	−0.02 (0.37)	−1.3 (0.26)	−0.05 (0.42)	−1.4 (0.34)	133.389	<0.001
Letters	6.0 (0.69)	6.3 (1.15)	6.0 (0.89)	6.0 (0.93)	0.576	0.632
Syllables	2.1 (0.36)	1.9 (0.67)	2.0 (0.37)	2.1 (0.37)	0.352	0.788
Arousal	3.1 (0.33)	3.0 (0.38)	3.1 (0.55)	3.1 (0.28)	0.793	0.500
Imageability	4.2 (0.96)	3.8 (1.51)	4.3 (1.21)	4.1 (1.24)	0.899	0.444
Bigram frequency	5265.97 (6442.04)	4419.183 (3848.24)	6273.18 (7628.60)	5381.85 (6407.67)	0.444	0.722
Ortho. Neighbors (*N*)	1.2 (1.09)	1.0 (1.59)	1.2 (1.10)	1.0 (1.31)	0.174	0.914
Frequency of *N* (FN)	19.1 (66.56)	38.3 (75.53)	62.6 (171.61)	29.6 (118.11)	0.771	0.513
Higher frequent *N* (HN)	0.3 (0.46)	0.4 (0.77)	0.5 (0.73)	0.6 (0.97)	0.635	0.594
Frequency of HN (FHN)	17.6 (65.79)	36.5 (73.90)	61.1 (171.38)	28.0 (118.31)	0.777	0.509
Frequency	15.7 (17.66)	17.1 (13.81)	12.1 (11.37)	13.8 (19.28)	0.554	0.646
**Response times (RT)**						
Total	750 (160)	742 (175)	727 (161)	739 (168)		
Control	752 (161)	748 (177)	730 (167)	750 (178)		
Patient	746 (159)	735 (173)	724 (155)	725 (154)		
**Error rate (ERR)**						
Total	4.3 (6.1)	4.4 (6.2)	3.3 (6.0)	5.5 (6.7)		
Control	3.1 (4.6)	3.3 (4.1)	2.1 (4.6)	4.1 (4.8)		
Patient	5.8 (7.4)	5.7 (8.1)	4.9 (7.2)	7.3 (8.3)		
**Response times (RT)**	lowFEAR	highFEAR	NEU	NEG	All Conditions
Total	747 (168)	734 (165)	739 (161)	741 (172)	740 (166)	
Control	751 (169)	741 (173)	742 (164)	750 (178)	746 (171)	
Patient	741 (166)	725 (154)	735 (157)	730 (164)	733 (160)	
**Error rate (ERR)**						
Total	4.36 (6.17)	4.43 (6.47)	3.83 (6.08)	4.97 (6.51)	4.40 (6.31)	
Control	3.25 (4.37)	3.12 (4.82)	2.61 (4.63)	3.76 (4.50)	3.19 (4.59)	
Patient	5.81 (7.73)	6.17 (7.85)	5.43 (7.30)	6.56 (8.22)	5.99 (7.77)	

##### fMRI Procedure

Before entering the scanner, participants received oral and written instructions to decide as quickly and accurately as possible via button press whether the presented letter string was a correct German word (left button) or a non-word (right button) of a button box. They were instructed not to press any button when presented with fillers. Eighteen practice trials (twelve words, six non-words, three null-events) that were not part of the stimulus set were used to familiarize the participants with the task. The stimuli were presented in an event-related design on a 32-inch LCD screen, specifically designed for use in an MRI environment (70). The screen resolution was 1,920 × 1,080 pixels, with a screen size of 69.8 × 39.3 cm, a refresh rate of 120 Hz, and a built-in linear luminance look-up table. The display was positioned at the far end of the bore and was viewed via a mirror positioned in the head coil of the MRI scanner. The total viewing distance was 220 cm. The total visible vertical extent of the screen subtended 10.2 degrees visual angle (deg). All stimuli were generated in MATLAB R2013a (Mathworks Inc., Natick, MA, USA) (71) using the Psychophysics toolbox (72). Stimuli were presented using Presentation software (73), which also recorded response times and accuracy of responses. Each trial began with the presentation of a fixation cross (+) in the center of the screen, which was presented for 2,148 ms on average (jitter: 1,401–4,098 ms), followed by the stimulus (800 ms) at the exact same position. The presentation of the words was randomized. The words were presented in black uppercase letters (Arial, font size 50) on a white background. Responses were given through a button box held in the right hand, the right button (blue) for a correct German word and the left button (green) for a non-word. An external pulse from the scanner controlled the start of the first trial.

##### fMRI Data Acquisition

fMRI data were acquired with a Siemens Magnetom Prisma-fit 3T MRI scanner with a 64-channel head/neck coil. The fMRI run for the lexical decision task consisted of 620 images and lasted about 10 min, including six dummy scans at the beginning. For distortion correction of the functional images an EPI fieldmap sequence based on the Siemens product fieldmapping sequence (TR 623 ms, TE1 4.92 ms, TE2 7.38 ms) was acquired. Structural imaging included a high-resolution T1w (TR 2400 ms, TE 2.24 ms) and T2w sequence (TR 3200 ms, TE 56 ms,), both with 0.8 mm isotropic resolution with sequences from the Human Connectome Project (HCP) Lifespan protocol (74). For preprocessing and statistical analysis, SPM12 software (75), running in a MATLAB R2013a environment (Mathworks Inc., Natick, MA, USA), and additional functions from AFNI (76) were used. Functional images were realigned, de-spiked (with the AFNI 3ddespike function), unwarped, and corrected for geometric distortions using the fieldmap of each participant and slice time corrected. The high resolution structural T1-weighted image of each participant was processed and normalized with the CAT12 toolbox (77) using default settings, each structural image was segmented into gray matter, white matter and CSF and denoised, then each image was warped into MNI space by registering it to the DARTEL template provided by the CAT12 toolbox via the high-dimensional DARTEL (60) registration algorithm. Based on these steps, a skull stripped version of each image in native space was created. To normalize functional images into MNI space, the functional images were co-registered to the skull stripped structural image and the parameters from the DARTEL registration were used to warp the functional images, which were resampled to 3 × 3 × 3 mm voxels and smoothed with a 6 mm FWHM Gaussian kernel. Statistical analysis was performed with a general linear model (GLM) two-staged mixed effects model. In the subject-specific first level model, each condition (discrete emotion: FEAR high/low, emotions dimension: Negativity yes/no) was modeled by convolving stick functions at its onsets with SPM12's canonical hemodynamic response function. Parameter estimates for each condition were calculated via these first-level GLMs, using a temporal high-pass filter (cutoff 128 s) to remove low-frequency drifts and modeling temporal autocorrelation across scans with an AR (1) process (78). For voxel-based group analyses, contrast images for effects of interest were calculated at the first level. These contrast images were used in second level analyses for a words vs. baseline contrast. All results from whole brain analyses are reported at a voxel-level threshold of *p* < 0.001 (uncorrected) with a FWE cluster-level correction of *p* < 0.05.

##### ROI Analysis

We performed three region of interest (ROI) analyses. The functionally ROI analysis was performed for our a priori regions (i.e., right amygdala, left and right inferior frontal gyrus) using the coordinates used by Briesemeister et al. (11). The amygdala and inferior frontal gyri were selected based on their suggested role in Panksepp's hierarchical emotion theory, representing secondary and tertiary level processes, respectively. The ROIs were created with a sphere of 6 mm in the right amygdala (x = 21, y = 2, z = −11) and in the left (x = −45, y = 35, z = 26) and right inferior frontal gyrus (x = 42, y = 26, z = −8). ROIs were built with the MarsBar toolbox implemented in SPM12. ROI extraction was performed with REX (79), based on the average contrast estimates of our four word conditions, for further statistical analysis.

#### Statistical Methods

Statistical analyses were performed in SPSS (version 18) (80) and R (version 4.0.5) (81). First, we defined primary, secondary and tertiary outcomes. Primary outcomes were any effects of activation in a 2 (group: JMEs/HCs) x 2 (discrete emotion: FEAR high/low) x 2 (emotions dimension: Negativity: yes/no) repeated measures ANOVA of our fMRI-data. Secondary outcomes were behavioral responses in the fMRI-paradigm (response times and error rates), again tested in a 2x2x2 design using a non-parametric ANOVA type test for repeated measure designs provided by the R package nparLD (82). *Post-hoc* contrasts in differences for the relative treatment effect were computed using a normal approximation with a Fisher transformation and the delta method, as described in Gunawardana and Konietschke (83). Mean lexical decision response times (LDRTs) were calculated for each participant and condition after the exclusion of non-responders, behavioral errors, and outliers, which were defined as responses faster than 300 ms or slower than 1,500 ms. In total 3.38 and 3.19% of responses were filtered for healthy controls and patients with JME, respectively. Error rates (ERRs) were calculated as the summed errors per condition and participant. Additionally, neuropsychological measures were defined as secondary outcomes. Group differences in neuropsychology variables were computed with univariate comparisons between groups, done via a non-parametric *t*-test (Brunner-Munzel Test) using the R package rankFD (84). Tertiary outcomes used the same model for behavioral responses as in the secondary outcomes but with an additional between subject factor for psychiatric comorbidities and the same model computed for patients only with type of seizure as a between subject factor (generalized tonic clonic seizures vs. absences and myoclonic seizures only vs. seizurefree). Further, we repeated the analysis of the secondary outcomes replacing group with type of antiseizure medication (mono- vs. polytherapy) only for patients with JME. We also conducted a repeated measures ANCOVA with 2 (discrete) x 2 (dimension) as within subject factors and duration of epilepsy as covariable only in patients, using the R-package nlme (85). In order to maintain control of the type I error and yet ensure adequate statistical power we employed the following scheme for *p*-value adjustment in our neuropsychological comparisons. For the primary outcomes, we used the Bonferroni procedure to control the FWER for each spherical regions of interest (i.e., multiplying the *p*-values by three). For secondary outcomes we used the Benjamini-Yekutieli (86) procedure to control the FDR at a level of 0.05. For tertiary outcomes, we did not conduct any correction for multiplicity as we considered them to be auxiliary analyses. For *p*-values we provide the unadjusted and adjusted version. Confidence intervals are unadjusted.

The effect measure used for primary outcomes was η*2* and for non-parametric analyses (secondary outcomes) the *RTE*. For comparisons between two groups *RTE* is the probability that a random subject from one group has a higher value in the outcome variable than a random subject from the other group. It is identical to the area under the curve when using the outcome variable to classify subjects into the two groups. For more than two groups it is the probability that a random subject from one group has a higher value in the outcome variable than a random subject from the total sample.

## Results

### Clinical Features

Twenty-eight patients (60.9%) and 12 controls (19.7%) presented a psychiatric disorder. 15.2% of patients had Axis I, 23.9% Axis II and 21.7% Axis I & II disorders (see Table 2). Considering multiple diagnoses in one patient, affective (15.2%), anxiety (13.0%), substance-related disorders (13.0%) and obsessive-compulsive personality disorders (OCD; 21.7%) represented the majority of psychiatric comorbidities. For a detailed description of psychiatric diagnoses see Supplementary Table 1. Due to multiple sessions, incompliance and tiredness of study participants, not all neuropsychological subtests have been performed in all patients (Supplementary Table 2).

**Table 2:** Descriptive statistics of sample.

	**Control group (*****n*** **=** **62)**				**JME patients (*****n*** **=** **47)**			
	***n* (%)**	**Min**	**Max**	**Mean (SD)**	***n* (%)**	**Min**	**Max**	**Mean (SD)**
**Sex**								
Female	33 (53.2%)				24 (51.1%)			
Male	29 (46.8%)				23 (48.9%)			
Age	62	18	62	27.71 (9.69)	47	14	52	27.09 (7.84)
Education in years	62	10	18	13.18 (1.94)	47	8	17	12.28 (2.10)
Epilepsy begin (age)				47	3	24	14.23 (3.83)
Epilepsy duration (in years)				47	0	34	12.85 (7.70)
**Seizure type (mulitple types in one patient)**								
GCTS				15 (31.9%)			
Absences				12 (25.5%)			
Myoclonus				29 (61.7%)			
Seizure free				16 (34.0%)			
**Medication**								
No Medication					3 (6.2%)			
Monotherapy					28 (58.3%)			
Polytherapy					17 (35.4%)			
**Medication (multiple medications in one patient)**								
Levetiracetam					34 (72.3%)			
Valproic acid					12 (25.5%)			
Lamotrigine					7 (14.9%)			
Zonisamide					2 (4.3%)			
Topiramate					1 (2.1%)			
Other					7 (14.9%)			
Any antidepressant 0 (0.0%)					5 (10.6%)			
**Psychiatric disorder (SCID 1&2)**								
No PD	49 (80.3%)				18 (39.1%)			
with PD	12 (19.7%)				28 (60.9%)			

*Shown are absolute numbers and percentages. PD, psychiatric disorders; SCID, Structured Clinical Interview for DSM-IV Disorders. One psychiatric diagnosis (SCID) missing in control and patient group*.

### Neuropsychology

In our non-parametric ANOVA type test (82) patients had significantly lower scores in the verbal intelligence test than healthy controls (*p* = 0.01). Patients with JME performed worse in single phonematic fluency (*p* = 0.01) as well as single (*p* = 0.06) and alternating semantic fluency (*p* < 0.001). Reading speed was decreased in patients with JME compared to healthy controls (*p* < 0.001). Despite having significantly lower scores than healthy controls, patients performed, for the most part, on average in all subtests. There were no differences in verbal memory functions: total learning score, delayed recall and recognition. All reported results were significant after correction for multiple comparisons. For a detailed description see Supplementary Table 2.

### Behavioral Results: Lexical Decision Response Times and Error Rates

A 2 (group: JMEs/HCs) x2 (discrete emotion: FEAR high/low) x2 (emotion dimension: Negativity: yes/no) non-parametric repeated measures ANOVA revealed a significant main effect of fear (*p* = 0.006) that was driven by faster responses of highFEAR words compared to lowFEAR words (detailed descriptive statistics are presented in Table 1, inferential statistics in **Table 4**, and in Supplementary Figure 1). Moreover, a FEAR x Negativity interaction approached significance (*p* < 0.001). Planned pairwise comparisons between all conditions revealed faster response times of highFEAR+NEU compared to lowFEAR+NEU words (*p* < 0.001). Error rates showed a significant main effect of group with healthy controls making significantly less errors (*p* < 0.001). A significant main effect of Negativity (*p* < 0.001) that was driven by an increased number of errors in response to words of negative valence (NEG) compared to words with neutral valence (NEU). Further, a FEAR x Negativity interaction (*p* < 0.001) was driven by more errors for highFEAR+NEG than highFEAR+NEU words. *Post-hoc* sensitivity analysis adding psychiatric comorbidity or type of seizure (generalized tonic-clonic seizures x myoclonus and/or absences x seizure free) did not change the significance of the aforementioned effects regarding response times and error rates. Further, no group or interaction with group was significant when comparing antiseizure medication (mono- vs. polytherapy) in patients only. Duration of epilepsy did not correlate with either, response time or error rate in the repeated measures ANCOVA.

### Neuroimaging Results

#### Whole Brain Analysis

**Figure 1:**
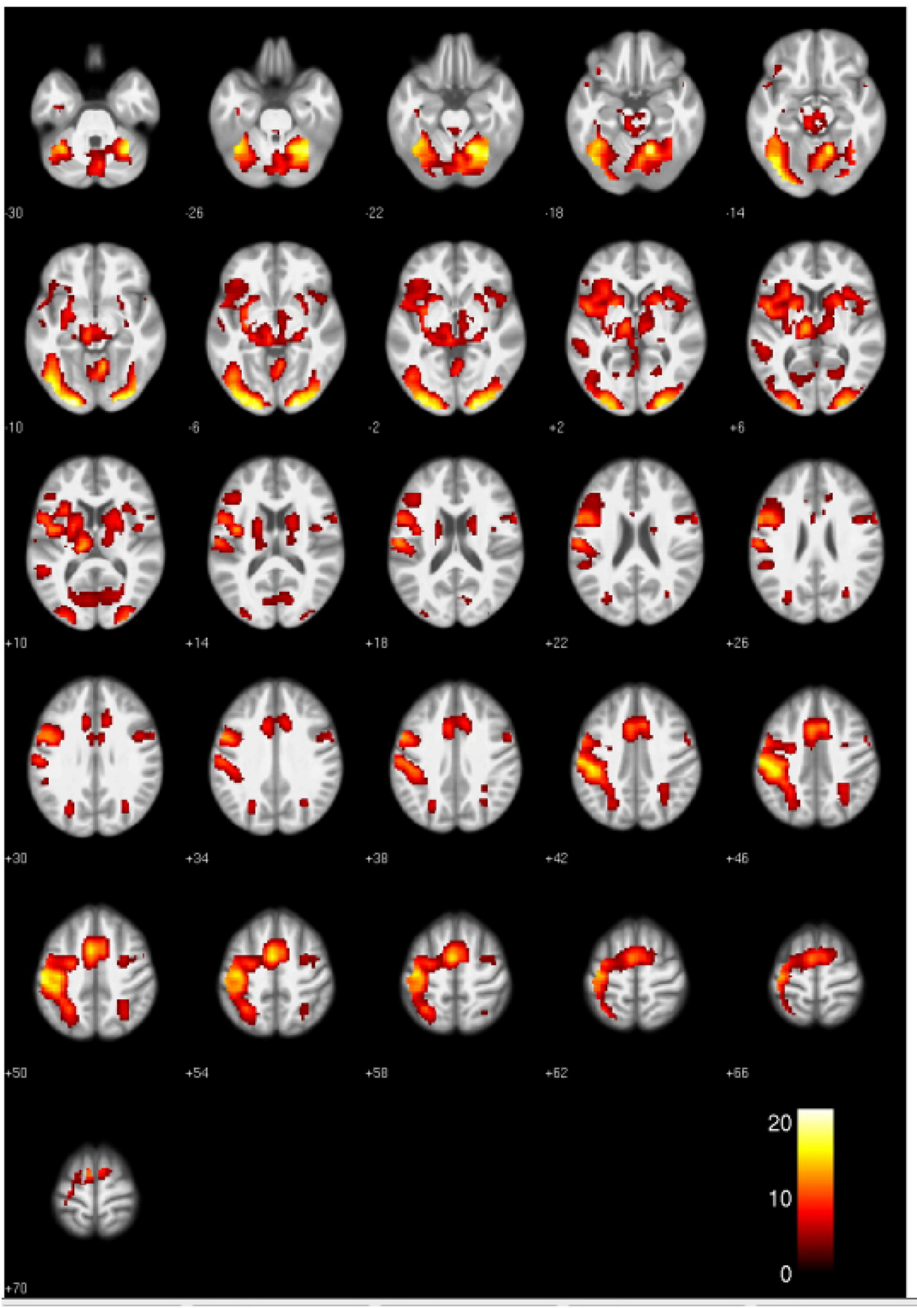
Activation clusters revealed by the whole brain analyses. Regions that elicited increased activation for words compared to baseline (irrespective of group) are shown in yellow. All clusters were extracted at a threshold of *p* < 0.001 [uncorrected, with a FWE cluster-level-correction (*p* < 0.05)].

The voxel-based whole-brain analysis across both groups revealed a main effect for words compared to fixation-baseline. Higher activity for words was found in a number of regions commonly involved in word processing, including an extended cluster in the left and right occipital lobe including inferior occipitotemporal, parietal and frontal areas, a left medial temporal cluster, a right occipitoparietal cluster, a right parahippocampal cluster and a cluster located in the brainstem. Please see Figure 1 and Table 3 for details. We did not observe any statistically significant differences between patients with JME and controls in the words vs. fixation-baseline contrast.

**Table 3:** Significant cluster of the whole brain analysis.

	**MNI coordinates**			**Volume (voxels)**	
**Region**	***x***	***y***	***z***		***T***
**Stimulus main effect**					
Words > baseline					
L middle temporal	−50	−44	10	196	7.40
R parietooccipital	28	−47	48	323	7.30
R parahippocampal	28	−2	50	129	6.23
Brainstem	0	−37	−38	70	5.52
**L/R posterior occipital, occipitotemporal, parietal, frontal**				14,851	
L occipitial	−20	−92	−10		21.50
R occipital	20	−92	−8		21.06
R inferior occipitotemporal	16	−92	−20		20.11
R fusiform gyrus	38	−54	−15		9.93
L primary somatosensory	−50	−20	48		18.05
L primary motor	−40	−4	12		14.21
R insula	33	18	5		8.84
R putamen	23	10	2		10.52
L pars opercularis	−54	6	25		12.49
R pars opercularis	33	18	5		7.64

*Data were extracted at a voxel-level threshold of p < 0.001 (uncorrected) and a cluster-level threshold (FWE) of p < 0.05*.

#### ROI Analyses

**Figure 2:**
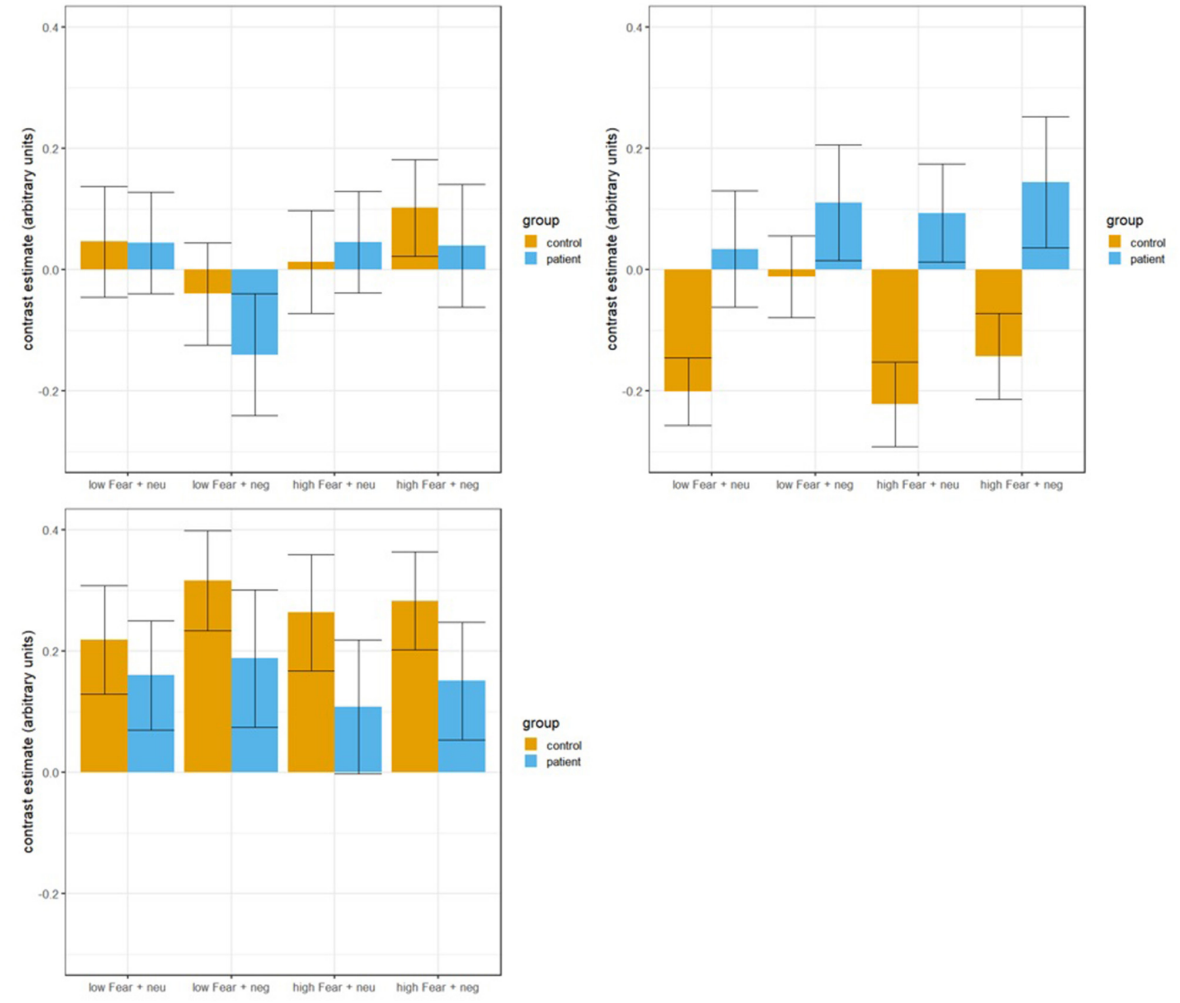
fMRI results for our conditions in the right amygdala (top left), right inferior frontal cortex (top right), and left inferior frontal gyrus (bottom left). Errror bars indicate standard error.

A 2 (group: JMEs/HCs) x 2 (discrete emotion: FEAR high/low) x 2 (affective dimension: Negativity yes/no) repeated measures ANOVA revealed a main effect of affective dimension in the right inferior frontal gyrus (*p* = 0.001), which was driven by higher activity in response to words with negative valence compared to words with neutral valence. The same repeated measures ANOVA in the right amygdala revealed a significant interaction of fear and negativity (*p* = 0.018), driven by higher activity in response to words with high fear values and negative valence compared to words with low fear values and negative valence. No differences were found in the left inferior frontal gyrus. A main effect of fear in the right amygdala (*p* = 0.06) driven by higher activity for words with high fear compared to low fear, as well as a main effect of group in the right inferior frontal gyrus (*p* = 0.06), driven by higher activity in patients with JME compared to healthy controls, did not reach significance after Bonferroni *p*-value correction. Please see Table 4 and Figure 2 for details.

**Table 4:** Statistical inference for the behavioral and fMRI data.

**Effect**								**χ^2^ - value**	***p* – value adjusted**	***RTE* difference**
**LDRT**										
Main Effect of FEAR							10.53	0.006	
FEAR x Negativity Interaction					12.42	<0.001	
lowFEAR + NEU > highFEAR + NEU					16.47	<0.001	0.047
lowFEAR + NEG < higFEAR + NEG					0.04	1	0.001
*ERR*										
Main effect of group							13.06	<0.001	
JME > HC								12.75	<0.001	0.135
Main effect negativity							15.63	<0.001	
FEAR x Negativity Interaction					11.64	<0.001	
lowFEAR + NEG > lowFEAR + NEU					0.10	1	0.009
highFEAR + NEG > highFEAR + NEU					30.31	<0.001	0.151
				MNI coordinates				
*Anatomical location (spherical ROI)*	L/R	BA	*x*	*y*	*z*	Size	*F*- value	*p* – value adjusted	η^2^ - value
**Main effect of negativity**										
Inferior frontal gyrus	R	47	42	26	−8	6 mm	13.46	0.001	0.112
Inferior frontal gyrus	L		−45	35	10	6 mm	2.10	0.450	0.019
Amygdala	R		21	2	−11	6 mm	1.90	0.513	0.017
Main Effect of FEAR									
Amygdala		R		21	2	−11	6 mm	5.59	0.060	0.050
FEAR x Negativity Interaction							
Amygdala		R		21	2	−11	6 mm	7.73	0.018	0.067
Main effect of group										
Inferior frontal gyrus		R	47	42	26	−8	6 mm	5.54	0.060	0.049
Inferior frontal gyrus		L		−45	35	10	6 mm	0.89	1	0.008
Amygdala		R		21	2	−11	6 mm	0.08	1	0.001

*Anatomical locations for selected main effects and interactions of negativity, fear and group. Anatomical p-values are adjusted for number of ROIs (N = 3). LDRT, response time in lexical decision task; ERR, error rate (in %) in lexical decision task; LDRTs and ERRs p-values are adjusted with the Benjamini-Yekutieli method, together with all other secondary variables*.

## Discussion

With this study we aimed to validate and to extend previous findings suggesting hierarchical processing of emotion (10, 11, 13). In support of our hypothesis, we found an interaction of discrete emotion and affective dimensional processing in the right amygdala, likely to reflect secondary level processing. Further, brain activation in the right inferior frontal gyrus points to affective dimensional processing, suggested to reflect tertiary level processing. The processing of emotional words was not influenced by psychiatric conditions, type of seizure or duration of epilepsy, as well as mono- vs. polytherapy in JMEs only. Patients and healthy controls did not show any differences in brain activity or response times, despite higher incidences of psychiatric conditions and slightly decreased verbal intelligence, verbal fluency and reading speed in patients with JME.

Based on the results of Briesemeister et al. (10, 11) and the idea that affectively conditioned stimuli like words access secondary and tertiary process levels within Pankespp's hierarchical emotion model (13), we expected the amygdala and the inferior frontal gyrus, to be involved in implicit emotional word processing (19, 22, 23, 87). Therefore, we tested healthy controls and patients with JME which are suggested to have cognitive, behavioral and emotional disturbances, related to subtle structural and functional alterations in frontal brain regions (44–47). Furthermore, we were interested in verbal and neuropsychological functions of patients with JME, and if psychiatric conditions, type of seizure, mono- vs. polytherapy, and duration of epilepsy were related to discrete emotion and affective dimensional processing. The whole brain analysis revealed brain regions typically activated in reading emotional words (22), involving occipital, temporal, parietal and frontal regions. Activity in the right amygdala in response to the emotional content of words, confirmed the results of Briesemeister et al. (11) and Nakic et al. (19), supporting the idea of an amygdala involvement in implicit emotion processing. According to Nakic et al. (19), amygdala activity indicates the processing of emotional salience, and regions relevant for behavioral responses such as the medial orbito-frontal gyrus and the anterior cingulate cortex receive input from the amygdala, thereby facilitating behavioral lexical decision responses. In support of this proposal, we observed an interaction of discrete emotion and affective dimension driven by higher activity in response to high fear words with negative valence compared to low fear words with negative valence in the right amygdala. In line with this pattern of activity, response times revealed a significant main effect of fear, driven by faster response times for high fear words compared to low fear words, and an interaction of discrete emotion and affective dimensional processing, driven by faster response times for low fear words with negative valence compared to low fear words with neutral valence. These behavioral interactions support Nakic et al. (19) findings, of a correlation between amygdala and anterior cingulate cortex activity in conditions showing enhanced word processing speed for negative words. Activity in the right inferior frontal gyrus revealed a main effect of negativity, driven by higher activity for negative compared to neutral words, which confirms Briesemeister et al's. results, and are line with the suggestion of the hierarchical emotion model of explicit evaluation of emotional salient stimuli on tertiary levels lending further support to Panksepp's proposal, that tertiary level processes “require expansive neocortical tissues that permit linguistic-symbolic transformations” (88). Thus, activity in the inferior frontal gyrus could reflect increased computational demands within the so-called *reading network* underlying semantic evaluation and integration processes of emotional information (20, 89–91).

However, the interaction of discrete emotion and affective dimensional processing in the amygdala in the current study is in contrast with Briesemeister's double dissociation of discrete emotion and affective dimension. Thus, it further challenges the amygdala's role in emotion processing. Briesemeister et al. (10, 11) suggested that activity in the inferior frontal gyrus in response to the affective dimension of positivity and the activity in the amygdala in response to the discrete emotion happiness reflect the dissociation of discrete emotion and affective dimensional processing in the brain. However, as the hierarchical emotion model focuses on primary process level emotions, the amygdala's role – as a potential secondary level process – is not yet clear, and the current results do not necessarily contradict the hierarchical emotion model. The observed activity in the amygdala could be based on the processing of biological relevant information and could reflect the interaction of top-down affective dimensional and bottom-up discrete emotion information carried by conditioned affective stimuli such as words (20, 92, 93).

The absence of significant differences in brain activity and response times between patients with JME and healthy controls during implicit emotion processing could be interpreted in favor of the notion that emotion processing in reading operates rather independently of other cognitive domains, as previously reported in emotional face recognition (94). However, these results were somewhat unexpected, as patients with JME were reported to present structural and functional differences in frontal regions, as well as neuropsychological deficits, related to executive functions (47). These results might be attributable to the implicit nature of the lexical decision paradigm and could point to independent processing of emotional information in reading, despite known altered explicit executive functioning in JME (37, 39, 40, 47). In addition, this result could suggest rather unaffected implicit emotion processing in patients with JME and could emphasize the importance of a differentiation of the implicit and explicit nature of tasks used in studying emotion processing, considering that most tasks used are of explicit nature (25–27, 29, 95–98). Error rates revealed a significant main effect of negativity, driven by higher error rates in response to negative as compared to neutral words, and an interaction, showing higher error rates in response to words with high fear values and with negative valence compared to words with high fear values and neutral valence. Further, we found a statistically significant difference in error rates between healthy controls and patients with JME. However, as the total number of errors was 3.19% (healthy controls) and 5.99% (patients with JME), respectively, we interpret this to be in a normal range and carefully interpret this difference to be due to impaired cognitive functions, as revealed by the results of the neuropsychological testing.

Neuropsychology revealed slower reading-speed, lower semantic and phonematic verbal fluency, and reduced verbal crystalline intelligence in patients with JME. Slower reading speed and comprehension extends on previous results found in patients with temporal lobe epilepsy and idiopathic epilepsies in children (99–101). Deficits in verbal fluency in our patients confirm existing results in patients with JME, pointing to disturbed executive processing supporting epilepsy-specific patterns of neuropsychological dysfunctions (99, 102). Reduced verbal intelligence might be interpreted as a constitute of reading ability and verbal executive functioning, as overall intelligence level is usually normal in this patient group (103, 104). Additionally, we found no significant association between psychiatric conditions, type of seizure disorder or epilepsy duration and the outcome of emotional word processing in the lexical decision task.

This study has some limitations related to the interpretation of neuropsychological data and brain activity in response to the emotional content of words. First, as psychiatric disorders in JME patients are reported to be increased, a special focus on the type of psychiatric disorder and a direct comparison between those may unveil additional information (105). For simplicity, we only controlled statistically for psychiatric disorders (yes or no) within our groups and their effect on affective processing. Second, as argued by Hamann (106), our fMRI-results might reveal deeper insights into brain mechanisms of implicit affective processing if we were to have presented more types of emotions than only fear/negativity in our paradigm. There are, however, some strengths that we like to highlight. This study validates and replicates the rationale of a previous study (11), using a different set of emotions. Due to our implicit task design and with the inclusion of patients with JME, we were able to unveil unaffected implicit affective processing in this patient group. To our knowledge, this is the first study to report results of implicit affective processing in this patient group, as most studies use paradigms which focus on explicit emotion processing (26, 29, 97, 98) (e.g., emotion recognition).

In this study, we showed an interaction of discrete emotion and affective dimensional processing in the right amygdala. This interaction was driven by higher activity in response to words with high fear values and negative valence, compared to words with low fear values and negative valence. We interpreted this activity to reflect the interaction of top-down and bottom-up circuits of primary and tertiary process levels in the amygdala, which is suggested to be involved in the conditioning of emotional stimuli in general. Higher activity in the right inferior frontal gyrus for negative words is in line with the results of Briesemeister et al. and was interpreted to signal explicit semantic evaluation and emotional memory integration on the tertiary process level. The results of the neuropsychological testing revealed subtle deficits in reading speed, phonematic and semantic fluency, and verbal intelligence in patients with JME compared to healthy controls. However, this did not result in any behavioral differences concerning discrete emotion and affective dimensional processing of patients with JME and healthy controls. Thus, the results of the current study could point to unimpaired implicit emotion processing of patients with JME during lexical decisions to words carrying emotional information.

## Data Availability Statement

The datasets presented in this article are not readily available because The decision of the Ethics Committee of the state of Salzburg does not include the sharing of the data. Requests to access the datasets should be directed to LR, lucas.rainer@stud.sbg.ac.at.

## Ethics Statement

The studies involving human participants were reviewed and approved by Salzburg Ethics Committee. Written informed consent to participate in this study was provided by the participants' legal guardian/next of kin.

## Author Contributions

LR, JH, GK, and MB contributed significantly to conception and design of the presented paper, acquisition, analysis and interpretation of the data as well as drafting of the paper. ES, PL, LK, SS-Y, MKi, MKr, GZ, and ET contributed to acquisition and analysis of data and revising the paper for intellectual content. GK, JH, MB, and ET contributed significantly to conception of the study, interpretation of the results and gave final approval of the submitted version of the manuscript. All authors contributed to the article and approved the submitted version.

## Funding

The presented research was funded by the FWF, Austrian Science Fund (Project Number: KLI 543 B-27).

## Conflict of Interest

ET reports personal fees from EVER Pharma, Marinus, Argenix, Arvelle/Angelini, Medtronic, Bial – Portela, S.A., NewBridge, GL Pharma, GlaxoSmithKline, Hikma, Boehringer Ingelheim, LivaNova, Eisai, UCB, Biogen, Genzyme Sanofi, GW Pharmaceuticals, and Actavis outside the submitted work; his institution received grants from Biogen, UCB Pharma, Eisai, Red Bull, Merck, Bayer, the European Union, FWF Osterreichischer Fond zur Wissenschaftsforderung, Bundesministerium fr Wissenschaft und Forschung, and Jubilaumsfond der sterreichischen Nationalbank outside the submitted work. GZ gratefully acknowledges the support of the WISS 2025 project; IDA-Lab Salzburg (20204-WISS/225/197-2019 and 20102-F1901166-KZP). The remaining authors declare that the research was conducted in the absence of any commercial or financial relationships that could be construed as a potential conflict of interest.

## Publisher's Note

All claims expressed in this article are solely those of the authors and do not necessarily represent those of their affiliated organizations, or those of the publisher, the editors and the reviewers. Any product that may be evaluated in this article, or claim that may be made by its manufacturer, is not guaranteed or endorsed by the publisher.
