# Supplementary material for: Emotional Word Processing in Patients With Juvenile Myoclonic Epilepsy
Source: Front Neurol. 2022 Jun 1;13:875950. doi: 10.3389/fneur.2022.875950 (PMC9201996; doi:10.3389/fneur.2022.875950)
Supplement: Supplementary file 1 [file Data_Sheet_1.docx]

**ONLINE Supplementary**

Table 1. Descriptive statistics of psychiatric comorbidities.

|  | **Control group (*n*=61)** | **JME patients (*n*=46)** |
| --- | --- | --- |
| **Axes** | *n* (%) | |
| No psychiatric disorder | 49 (80.3%) | 18 (39.1%) |
| with psychiatric disorder | 12 (19.7%) | 28 (60.9%) |
| Axis I disorder only | 7 (11.4%) | 7 (15.2%) |
| Axis II disorder only | 4 (6.5%) | 11 (23.9%) |
| Axis I & II disorder | 1 (1.6%) | 10 (21.7%) |
| *Current* DSM-IV *diagnoses, multiple diagnoses in one patient* |  |  |
| Axis I disorders |  |  |
| Affective disorders (296.2x, 296.3x, 293.83) | 3 (4.9%) | 7 (15.2%) |
| Schizophrenia and other psychotic disorders (298.8, 295.30, 293.81) | 1 (1.6%) | 2 (4.3%) |
| Substance-related disorders (303.90, 304.30, 304.80) | 4 (6.5%) | 6 (13.0%) |
| Anxiety disorders (300.02, 300.21, 300.01, 300.22, 300.29, 300.23, 300.3, 309.81, 308.3) | 2 (3.3%) | 6 (13.0%) |
| Eating disorders (307.1, 307.51, 307.50) | 0 (0%) | 2 (4.3%) |
| Adjustment disorders (309.9, 309.24, 309.0) | 3 (4.9%) | 4 (8.7%) |
| Axis II disorders |  |  |
| *Cluster A* |  |  |
| Paranoid personality disorder (301.00) | 0 (0%) | 4 (8.7%) |
| Schizotypal personality disorder (301.22) | 1 (1.6%) | 0 (0%) |
| *Cluster B* |  |  |
| Antisocial personality disorder (301.7) | 1 (1.6%) | 5 (10.9%) |
| Borderline personality disorder (301.83) | 2 (3.3%) | 4 (8.7%) |
| Histrionic personality disorder (301.50) | 0 (0%) | 3 (6.5%) |
| Narcissistic personality disorder (301.81) | 0 (0%) | 3 (6.5%) |
| *Cluster C* |  |  |
| Avoidand personality disorder (301.82) | 1 (1.7%) | 4 (8.7%) |
| Dependent personality disorder (301.83) | 0 (0%) | 2 (4.3%) |
| Obsessive-compulsive personality disorder (301.4) | 1 (1.7%) | 10 (21.7%) |
| *Personality disorder not otherwise specified* |  |  |
| with depressive personality traits (301.9) | 0 (0%) | 3 (6.5%) |
| with passive-aggressive personality traits (301.9) | 0 (0%) | 2 (4.3%) |

Table 2. Secondary statistics: Neuropsychology. Performance in verbal intelligence, verbal fluency, verbal memory, reading speed.

|  | **Control group** | | | |  | **JME patients** | | | |  | **Effect size** | |  | **Significance test** | | | |
| --- | --- | --- | --- | --- | --- | --- | --- | --- | --- | --- | --- | --- | --- | --- | --- | --- | --- |
|  | *n* | Min | Max | Mean (SD) |  | *n* | Min | Max | Mean (SD) |  | *RTE* | 95% CI |  | *t* -value | df | *p* - value | *p* - value adjusted |
| *Verbal Intelligence* | |  |  |  |  |  |  |  |  |  |  |  |  |  |  |  |  |
| MWT-B | 61 | 15 | 35 | 28.00 (3.88) |  | 42 | 17 | 35 | 25.76 (3.97) |  | 0.670 | (0.561- 0.779) |  | 3.113 | 83.391 | 0.002 | 0.01 |
| *Verbal Fluency* |  |  |  |  |  |  |  |  |  |  |  |  |  |  |  |  |  |
| RWT Phonemic - single (S) | 61 | 8 | 41 | 24.95 (7.17) |  | 42 | 6 | 45 | 20.38 (7.87) |  | 0.675 | (0.567- 0.784) |  | 3.215 | 82.483 | 0.002 | 0.01 |
| RWT Phonemic - switch (G-R) | 61 | 8 | 34 | 21.62 (4.91) |  | 42 | 4 | 42 | 18.85 (7.33) |  | 0.650 | (0.534- 0.766) |  | 2.587 | 64.995 | 0.012 | 0.057 |
| RWT Semantic - single (animals) | 61 | 19 | 62 | 36.90 (9.37) |  | 42 | 14 | 55 | 31.07 (9.37) |  | 0.686 | (0.578- 0.795) |  | 3.409 | 83.241 | 0.001 | 0.006 |
| RWT Semantic - switch (sport - fruit) | 61 | 16 | 40 | 23.97 (4.05) |  | 42 | 7 | 33 | 20.38 (5.08) |  | 0.727 | (0.620- 0.835) |  | 4.233 | 63.865 | < 0.001 | <.001 |
| *Verbal Memory* |  |  |  |  |  |  |  |  |  |  |  |  |  |  |  |  |  |
| VLMT Total learning | 61 | 40 | 71 | 56.63 (7.17) |  | 44 | 11 | 64 | 53.15 (9.18) |  | 0.591 | (0.481- 0.701) |  | 1.640 | 99.928 | 0.104 | 0.467 |
| VLMT Delayed recall | 61 | 6 | 15 | 12.26 (2.26) |  | 44 | 3 | 15 | 11.77 (2.70) |  | 0.558 | (0.446- 0.670) |  | 1.026 | 94.660 | 0.307 | 1 |
| VLMT Recognition | 61 | 8 | 15 | 13.88 (1.45) |  | 44 | 5 | 15 | 13.59 (1.88) |  | 0.543 | (0.434- 0.653) |  | 0.790 | 88.770 | 0.431 | 1 |
| *Reading Speed* |  |  |  |  |  |  |  |  |  |  |  |  |  |  |  |  |  |
| Reading test | 61 | 28 | 75 | 51.22 (9.83) |  | 41 | 5 | 63 | 41.90 (12.49) |  | 0.721 | (0.617- 0.825) |  | 4.221 | 76.902 | <0.001 | <.001 |

MWT-B, Mehrfach-Wortschatz-Intelligenztest. RWT, Regensburger Wortflüssigkeitstest. VLMT, Verbaler Lern- und Merkfähigkeitstest. RTE, relative treatment effect. *p*-values are adjusted with the Benjamini-Yekutieli method.


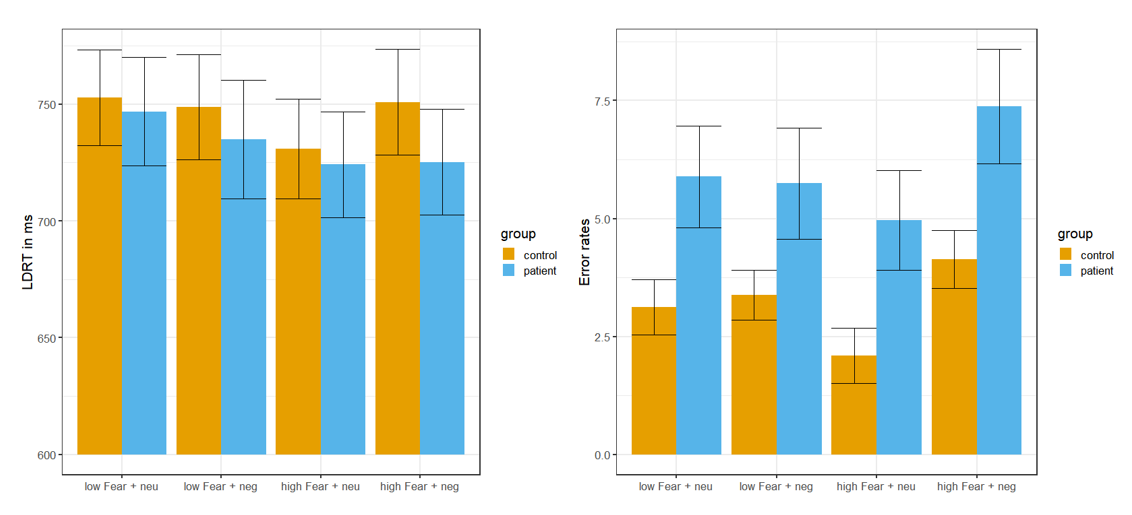


Figure 1. Behavioral lexical decision performance. Depicted are the mean lexical decision response times (LDRTs), in milliseconds, and the mean summed error rates per group and condition. Error bars indicate standard error.
